# Therapeutics

**Published:** 1876-04

**Authors:** 


### V. THERAPEUTICS.

1. *Diabetes Treated With Glycerine.* JULIUS JACOBS. (*Virchow's Arch.; Allg. Med. Centralz.*, 1876, No. 9.)

Like many other remedies, glycerine has been recommended for diabetes at different times; of late, Prof. Schultzen advocated its use very strongly, and in support of his recommendation advanced the following theory: Diabetic patients lose by the excretion of sugar an immense amount of respiratory food, and therefore must use up their fat and albumen in its stead. If they receive glycerine which within the body is not transformed to sugar, but directly to carbonic acid and water, the respiratory machine can be fed without waste of the tissues.

The author having two severe cases of diabetes tested the influence of glycerine on this disease. During the whole time of observation the two patients received at certain intervals a fixed quantum of mixed food, so that the daily ratio was alike throughout the whole course; and each took in 24 hours the following mixture: Glycerine, 25 grammes; tartar. acid, 5 grammes; water, 700 grammes.

In both cases he observed a gradual decrease in the quantity of urine, and a proportionate decrease in the quantity of water the patients drank, without their having diarrhœa. The daily quantity of sugar was reduced to a certain small amount while the patients, gradually and steadily, gained in strength, so much so that after a short time both patients did not feel any signs of their sickness. While the one patient did not gain much in weight, though certainly he did not lose any, the other added 4,400 grammes to his bodily weight within three months. The patients always had a good appetite without that annoying sensation of hunger so troublesome to these patients; they never had diarrhœa or vomiting.

No remedy has yet been known which could completely cure the diabetes; but some remedies are known to moderate the most prominent symptoms (apparent cure) and most likely to prolong the life of the patient. And among these remedies glycerine stands uppermost because taken continuedly in the above doses, it causes a marked improvement, at least for awhile, and it does it even when the patient is allowed to live on mixed diet.

2. *Salicylic Acid for Articular Rheumatism.* KATZ. (*Deutsche Med. Wochenschr.*, 1876, No. 4.)

To confirm the beneficial influence of the salicylic acid upon rheumatic arthritis (see Summary March No. of this vol.), Katz reported the following case: A baker was laid up with articular rheumatism on Jan. 4; at first the ankles, then the knees, later the fingers, wrists and shoulders were affected; great tenderness, soreness and swelling of the joints, fever moderate, pulse 100 to 120; ordered nitrate of potass., quinine and morphine at night. No improvement; on the evening of the 14th, temper., 39.3° C., pulse 100, both shoulder joints exceedingly tender, both wrists greatly swollen, the fingers thick and immovable; patient took one gramme (about 15 grains) of salicylic acid in a wafer at six P. M., and half a gramme every hour afterward till midnight, taking altogether four grammes (one drachm) of salicylic acid. When the doctor called next morning he was greatly surprised by finding his patient out of the bed and using his hands and arms quite freely. Took two grammes of the acid during the afternoon; in the evening no fever, pulse 90, no pain, no swelling of joints; patient was up, complaining of nothing except a profuse perspiration. Forty-eight hours after the beginning of the treatment with salic. acid he could walk alone.

3. *Treatment of Erysipelas.* PROF. D. W. YANDELL. (*Louisville Med. News*, Feb., 1876.)

After reviewing cursorily the treatment of this disease by Gross, Quain, Elliotson, Velpeau, Malgaigne and others, the Professor gives, in one of his excellent clinical lectures, his treatment of this disease, which is here epitomized. Local remedies are not highly flattered by the lecturer. The only "grateful" external application mentioned (unenthusiastically, however,) is the benzoated oxide of zinc ointment with carbolic acid, one grain of the latter to ten or twenty of the former. A closely applied roller bandage, in some cases kept moist by warm or cold lotions, is recommended when the extremities are the seat of the disease. If incisions are thought best, they should not be more than an inch long, or, at most, two or three inches long, carried along the direction of subjacent muscular fibre, deep enough to reach the matter. For œdema, simple lancet punctures are sufficient. In simple cases, in patients of "fair constitutions," an emetic should be administered, followed by a mercurial purge, combined, if necessary, with an opiate. In many cases naught more is needed. If, subsequently, the disease persist, then cooling remedies will be needed. If debility ensue ultimately, the general treatment should be tonics and stimulants. Food should be given in small quantities, and of the blandest kind. Muriated tincture of iron is a specific with many physicians, but not with the Professor. After a protracted, methodical trial of this agent in erysipelas, along with the late Professor Rogers, the conclusion reached by these two gentlemen was, that they were "not able to see any special good from it, except in cases where tonics were clearly indicated, and in those cases, occasionally met, where *albumen was found in the urine.*" In the latter class of cases, the iron possesses *peculiar efficacy.* "Indeed, it seemed that the power of the muriated tincture was just in proportion to the amount of albumen contained in the urine." Quinine is unequivocally good in two classes of cases only; *first,* in those occurring in malarial districts or malarial seasons; *second,* in those where the septic element is greatly in the ascendant, as in traumatic examples of the disease. In the former it will seldom disappoint, and in the second it is "well nigh the only ground of hope." No *specific* treatment exists, hence none is given by the Professor.

4. *Salicylic Acid for Foul Breath and Offensive Expectoration.* Prof. DA COSTA. (*Phila. Med. and Surg. Reporter*, Feb., 1876.)

The following prescription, used as a mouth wash and gargle, has yielded much satisfaction in the above named troubles:

R. Sodii Boratis, Acidi Salicylici, *aa*, grs. x; Glycerinæ, dr. j; Aquæ, ad oz. j. M.

The Professor recommends this combination in cases of foul breath arising from almost any cause whatever, as from fetid bronchitis, from disordered stomach, or indigestions with bad breaths from pulmonary abscess or cancer, etc., etc. Five grains, three times a day, were given; The purification of the breath was accomplished satisfactorily; the expectoration was improved in odor, but was not abolished.

5. *The Physiological Action of Prussic Acid.* BOEHEN and KNIE. (*Allg. Med. Centralz.*, 1876, No. 10.)

The authors arrived at the following conclusions:

1. The prussic acid has a direct influence on the centres of the nervous system, and in great doses annihilates their functions.

2. The disturbances of respiration and circulation are caused by a stimulation of the medulla through the poison.

3. The pneumogastric nerve is not implicated.

4. Atropia can not be regarded as an antidote against prussic acid; and in case of poisoning, artificial respiration is the only reliable remedy.

6. *Subcutaneous Injection of Lamb's Blood in Cases of Melancholia Considered Incurable.* VOISIN. (*Gazette des Hôpitaux*, No. 5.)

A patient is described whose grave condition was not ameliorated by the most tonic regimen. V. opened the jugular vein of a lamb by a longitudinal incision and withdrew into a porcelain vessel fifty grammes of blood. He then introduced a needle into the internal face of the right arm of his patient, gave it a lateral motion so as to tear open the lymphatics and capillaries, filled with blood a glass syringe terminating with a rubber tube which was fitted to the needle, and injected the blood without difficulty. A tumor as large as an apple resulted, the patient complaining of slight pain. A twisted suture was applied to the wound in the lamb. There was slight inflammation during the next few days, which soon disappeared. In five days the tumefaction was gone, and the general condition was improved. A second injection of forty-five grammes of blood followed. No evil results. As there was no albuminuria and no bile pigment in the urine, it followed that the injected blood globules remained in the system. The urine, heated and treated with nitric acid, exhibited a slight color of the lees of urine. The general condition continued to improve. The subcutaneous injections of morphia, which were not well borne before the injection of blood, were soon more and more tolerated.

Voisin has experimented in a second case with similar measures—introducing the blood every eight days with satisfactory results.

7. *Treatment of Scrofulous Sores or Runnings.* McLennan. (*Med. Times and Gaz.*, 1876.)

The writer, in a communication to the *Times and Gazette*, states that for the past twelve or thirteen years he has treated the above-named troubles with corrosive-sublimate in whisky. Three grains of this salt put into a pint bottle of whisky, is the strength of the solution used. A rag dipped in this preparation twice or thrice a day and laid on the sore and continued till the sores are all dried up, is all that is needed. In no case in which this treatment has had a fair trial, has it failed.

8. *A General Antidote for Poisons.* Jeannel. (*Medical Press and Circular.*)

M. Jeannel gives the following formula for an antidote for a number of deadly poisons:

| | | |
|---|---|---|
| Solution of Sulphate of Iron (D. 1.45), . . . | 100 | parts. |
| Water, . . . . . . . . . . . . | 800 | " |
| Calcined Magnesia, . . . . . . . | 80 | " |
| Washed Animal Charcoal, . . . . . . | 40 | " |

These ingredients are kept separate, the solution of sulphate of iron in one vessel, the magnesia and charcoal in another, with some water. When needed, the sulphate solution is poured into the last mentioned receptacle and violently agitated. The mixture should be administered promptly in doses of from 1.6 to 3.3 ounces. From experiments, M. Jeannel finds that this antidote, employed in proper proportions, renders preparations of arsenic, zinc and digitalin completely insoluble.

9. *Dressing for Burns.* Dr. Q. C. Smith, of Cloverdale, Cal. (*Pacific Med. and Surg. Journal.*)

Mix subnitrate of bismuth with pure honey till it forms a thick paste and spread it over the burn and contiguous parts plentifully and cover with cotton wool batting, and bind closely. Remove the dressing in three or five days by soaking in water, and reapply same remedy. The reporter considers, after trying many kinds of dressing, that this dressing is the best.

10. *Ergot of Rye as an Antipyretic.* Hayem. (*Rev. de Therap.; The Clinic.*)

The writer tried ergot in cases of enteric fever, with the object of lowering the temperature. The results were very satisfactory, and its employment in this disease seems to M. Hayem preferable to that of quinia or digitalis. Under ergot there is a much more rapid defervescence; and at the period of the acme, instead of there being a rise in the temperature chart, a plateau is obtained. In some cases in which the ergot was only given during the day, the evening temperature was not so high as the morning. The dose varied from thirty to fifty grains in the twenty-four hours.

11. *Temperature of Drunkards.* Reincke. (*Deutsche Arch. f. Klin. Med.*, 1875.)

Drunkenness—profound—can be easily diagnosticated from other causes of coma by the fact that in the former the temperature is diminished. The internal temperature of intoxicated persons exposed to cold may fall to an extent truly astonishing. Rectal temperature will commonly fall to 95° or 93° F. Even 82° F. has been annotated, and in one instance the temperature fell to 75 2° F., and the subject upon whom this extraordinary temperature was noticed regained his normal heat in twenty-three hours.

